# Xiangdan Injection attenuates experimental sepsis-induced myocardial injury by suppressing inflammation and preserving mitochondrial ultrastructure

**DOI:** 10.3389/fphar.2026.1860982

**Published:** 2026-08-04

**Authors:** Yan Qian, Wenmin Yang, Chunmin Zhang, Jinda Huang, Qiyi Zeng, Yuan Zhou

**Affiliations:** 1 Department of Emergency Intensive Care Unit, Wuhu Hospital, East China Normal University, Wuhu, Anhui, China; 2 Department of PICU, Guangzhou Women and Children’s Medical Center, Guangzhou Medical University, Guangzhou, Guangdong, China; 3 Department of Pediatrics, Zhujiang Hospital, Southern Medical University, Guangzhou, Guangdong, China; 4 Outpatient Clinic, The Affiliated Chuzhou Hospital of Anhui Medical University (The First People’s Hospital of Chuzhou), Chuzhou, Anhui, China

**Keywords:** cardioprotection, mitochondrial dysfunction, sepsis-induced myocardial injury, systemic inflammation, xiangdan injection

## Abstract

**Background:**

Sepsis-associated cardiac dysfunction is a major contributor to morbidity and mortality and is driven by dysregulated inflammation and impaired cellular bioenergetics. Therapeutic strategies that target both processes remain limited.

**Methods:**

This study evaluated the cardioprotective effects of Xiangdan Injection in murine sepsis models induced by Gram-positive *Staphylococcus aureus* and Gram-negative *Escherichia coli*.

**Results:**

Sepsis caused reduced survival, myocardial structural damage, increased circulating inflammatory mediators, elevated cardiac injury biomarkers, and mitochondrial ultrastructural disruption. Xiangdan Injection improved survival in a dose-dependent manner (p < 0.05), reduced serum IL-6, TNF-α, IL-1β, and HMGB1 levels (all p < 0.05), and decreased BNP, CK-MB, and cardiac troponin I levels (all p < 0.05). Transmission electron microscopy showed preservation of mitochondrial ultrastructure, reflected by reduced mitochondrial damage scores (p < 0.05).

**Conclusion:**

These findings indicate that Xiangdan Injection attenuates sepsis-induced myocardial injury by suppressing systemic inflammation and preserving mitochondrial structure. Comparable effects were observed in both bacterial models, supporting further investigation of Xiangdan Injection as a potential adjunctive intervention for sepsis-associated myocardial injury.

## Introduction

Sepsis remains a major global health challenge and a leading cause of mortality in critically ill patients, characterized by a dysregulated host response to infection that culminates in life-threatening organ dysfunction (Santacroce et al., 2024; La Via et al., 2024). Among the affected organs, the heart is particularly vulnerable, and sepsis-induced myocardial injury (SIMI) has emerged as a critical determinant of disease severity and clinical outcome (Caraballo and Jaimes, 2019). Increasing evidence indicates that myocardial dysfunction in sepsis is not merely a secondary consequence of systemic illness but a central component of the pathological process contributing to multi-organ failure (Chang et al., 2025).

The pathophysiology of SIMI is complex and involves the convergence of inflammatory dysregulation, metabolic disturbances, and structural cellular damage. Excessive release of pro-inflammatory cytokines, including interleukin-6 (IL-6), tumor necrosis factor-α (TNF-α), and interleukin-1β (IL-1β), disrupts myocardial contractility through impairment of calcium homeostasis, induction of oxidative stress, and endothelial dysfunction. In addition, high-mobility group box 1 (HMGB1), a late-phase inflammatory mediator, perpetuates the inflammatory response and exacerbates myocardial injury (Zhao et al., 2022; Chen et al., 2017; Ren et al., 2026). Alongside these inflammatory processes, mitochondrial dysfunction has been increasingly recognized as a pivotal driver of septic cardiomyopathy. Structural disruption of mitochondrial membranes and cristae impairs ATP production, promotes reactive oxygen species generation, and activates apoptotic pathways, thereby contributing to cardiomyocyte dysfunction and death (Luís et al., 2022; Brandquist and Kielian, 2025; Guarino et al., 2025).

Despite advances in critical care management, including hemodynamic support and antimicrobial therapy, current treatment strategies for septic cardiac dysfunction remain largely supportive and fail to directly target the underlying molecular mechanisms. This therapeutic gap underscores the need for novel interventions capable of simultaneously modulating inflammatory responses and preserving mitochondrial function (Prieto and Schinella, 2022).

Xiangdan injection is a traditional Chinese medicinal preparation derived from *Salvia miltiorrhiza* Bunge [Lamiaceae; *Salviae miltiorrhizae radix et rhizoma*] (Danshen) and *Dalbergia odorifera* T.C.Chen [Fabaceae*; Dalbergiae odoriferae lignum*] (Jiangxiang), two medicinal materials widely used in the management of cardiovascular and circulatory disorders. Previous experimental and clinical studies have reported anti-inflammatory, antioxidative, and cardioprotective activities associated with Xiangdan injection and related Danshen-containing formulations, supporting their potential therapeutic value in inflammatory cardiovascular conditions (Prieto and Schinella, 2022; Li et al., 2021).

Moreover, most preclinical investigations of sepsis rely on single-pathogen models, whereas clinical sepsis encompasses a heterogeneous spectrum of microbial etiologies. Whether therapeutic interventions demonstrate consistent efficacy across both Gram-positive and Gram-negative infections is an important yet underexplored question with significant translational implications (Dyck et al., 2024).

Therefore, the present study aimed to systematically evaluate the cardioprotective effects of Xiangdan injection in murine models of sepsis induced by both *Staphylococcus aureus* and *Escherichia coli*. We hypothesized that Xiangdan injection would attenuate myocardial injury through coordinated suppression of inflammatory signaling and preservation of mitochondrial integrity across both Gram-positive and Gram-negative sepsis models.

The present study extends previous investigations by evaluating the effects of Xiangdan injection across both Gram-positive and Gram-negative sepsis models while integrating inflammatory, biochemical, histopathological, and mitochondrial ultrastructural assessments to explore cardioprotective responses across both Gram-positive and Gram-negative sepsis models.

## Materials and methods

### Animals

Animals were assigned to nine experimental groups (n = 10 per group), including a sham control group, untreated sepsis groups, and Xiangdan-treated groups receiving three dosage levels (2.0, 2.5, and 5.0 mL/kg) in both Gram-positive (*S. aureus*) and Gram-negative (*E. coli*) sepsis models.

Male C57BL/6 mice (8–10 weeks old, body weight 22–25 g) were used for all experiments. Animals were housed under specific pathogen-free (SPF) conditions with controlled environmental parameters (temperature: 22 °C ± 2 °C; relative humidity: 55% ± 10%; 12 h light–dark cycle) and provided *ad libitum* access to standard chow and water.

## Experimental design and group allocation

Animals were randomly assigned to nine experimental groups (n = 10 per group), including a sham control group, untreated sepsis groups, and Xiangdan-treated groups at three dosage levels (2.0, 2.5, and 5.0 mL/kg) for both Gram-positive and Gram-negative sepsis models. Randomization was performed to minimize allocation bias, and all analyses were conducted in a blinded manner where applicable.

### Sepsis induction

Experimental sepsis was induced by intraperitoneal injection of either *S. aureus* (ATCC 25923) or *E. coli* (ATCC 25922). Bacterial strains were cultured overnight in Luria–Bertani broth at 37 °C under aerobic conditions. Cultures were harvested during the logarithmic growth phase, centrifuged, and resuspended in sterile saline. Bacterial concentrations were adjusted to 1 × 10^8^ CFU/mL, and the administered dose was confirmed by colony-forming unit (CFU) assays. The selected inoculum was based on preliminary optimization and previous studies to induce a reproducible sepsis phenotype with moderate mortality and systemic inflammatory response (Hoerr et al., 2012).

### Model validation

Successful establishment of the sepsis model was confirmed based on a combination of clinical, biochemical, and histopathological criteria. Following bacterial challenge, animals exhibited characteristic signs of sepsis, including reduced activity, piloerection, impaired responsiveness, and altered respiratory patterns. In addition, significant elevations in systemic inflammatory markers (IL-6, TNF-α, IL-1β, and HMGB1) and cardiac injury biomarkers (BNP, CK-MB, and cardiac troponin I) were observed in untreated sepsis groups compared with sham controls. Histopathological analysis further demonstrated marked myocardial structural disruption, including inflammatory infiltration, interstitial edema, and cardiomyocyte disorganization. These findings confirmed successful induction of sepsis-associated myocardial injury in both Gram-positive and Gram-negative models.

## Xiangdan Injection treatment protocol

Xiangdan Injection (Xiangdan Zhusheye, 香丹注射液; Batch No. XDJ-0617) was obtained as a commercially manufactured sterile injectable traditional Chinese medicine preparation from Guangzhou Baiyunshan Mingxing Pharmaceutical Co., Ltd (Guangzhou, China). The formulation is derived from *S. miltiorrhiza* Bunge [Lamiaceae*; Salviae miltiorrhizae radix et rhizoma*] and *D. odorifera* T.C.Chen [Fabaceae*; Dalbergiae odoriferae lignum*], medicinal materials recognized in the Chinese Pharmacopoeia. The preparation was supplied as a ready-to-use pharmaceutical product and was used directly without additional extraction, fractionation, purification, or chemical modification by the investigators.

Information relevant to the ConPhyMP (Consensus Statement on the Phytochemical Characterisation of Medicinal Plant Extracts) and GA Best Practice recommendations is provided in Supplementary Tables S1, S2.

Xiangdan injection was administered intraperitoneally at doses of 2.0, 2.5, and 5.0 mL/kg according to group allocation. To facilitate dose interpretation, published quality-control data for commercially available Xiangdan Injection formulations report concentrations of tanshinone IIA (0.27 mg/mL) and salvianolic acid B (1.03 mg/mL). Based on these reported values, the administered doses of 2.0, 2.5, and 5.0 mL/kg correspond approximately to 0.54, 0.68, and 1.35 mg/kg tanshinone IIA and 2.06, 2.58, and 5.15 mg/kg salvianolic acid B, respectively.

Treatment was initiated 1 h prior to sepsis induction and repeated immediately after bacterial challenge in order to ensure adequate drug exposure during the early phase of sepsis, when inflammatory activation and mitochondrial injury are rapidly triggered. The selected doses were based on previously published pharmacological studies employing Xiangdan Injection in rodent models and were chosen to evaluate potential dose–response relationships in experimental sepsis. The calculated exposures of representative marker constituents remained within the low mg/kg range, supporting the pharmacological plausibility of the selected dose levels and reducing the likelihood of non-specific high-dose effects. This design was intended to assess the mechanistic cardioprotective potential of Xiangdan injection under controlled experimental conditions. The dosing regimen and route of administration were selected based on previous experimental studies reporting systemic anti-inflammatory and cardioprotective effects of Xiangdan injection in sepsis and cardiovascular models, which employed comparable dosing ranges and intraperitoneal delivery (Chang et al., 2025; Prieto and Schinella, 2022).

Mitochondrial structural integrity was evaluated using the Flameng scoring system, a semi-quantitative ultrastructural grading method based on transmission electron microscopy. This scoring system classifies mitochondrial damage on a scale from 0 to 4, where 0 indicates normal mitochondrial structure, one indicates mild swelling with preserved cristae, two indicates moderate swelling with partial cristae disruption, three indicates severe swelling with significant cristae loss, and four indicates complete structural disruption with loss of membrane integrity. Scoring was performed based on cristae organization, membrane integrity, and mitochondrial swelling (Flameng et al., 1980).

### Clinical monitoring and survival assessment

Following sepsis induction, animals were monitored at regular intervals for clinical signs, including activity level, posture, respiratory pattern, and responsiveness. Survival was recorded up to the experimental endpoint (12 h). The 12-h observation period was selected to capture the acute phase of sepsis progression, during which rapid inflammatory activation, myocardial injury, mitochondrial damage, and early mortality are prominently observed in murine sepsis models. Animals reaching predefined humane endpoints were euthanized under deep anesthesia. Animals were anesthetized with sodium pentobarbital (50 mg/kg, intraperitoneal injection) prior to euthanasia, ensuring complete loss of reflexes before cervical dislocation.

### Blood collection and biochemical analysis

Serum concentrations of inflammatory cytokines, including interleukin-6 (IL-6), tumor necrosis factor-α (TNF-α), interleukin-1β (IL-1β), and high-mobility group box 1 (HMGB1), as well as cardiac injury biomarkers including B-type natriuretic peptide (BNP), creatine kinase-MB (CK-MB), and cardiac troponin I (cTnI), were quantified using commercially available enzyme-linked immunosorbent assay (ELISA) kits, according to the manufacturers’ instructions).

### Histopathological analysis

**FIGURE 1:**
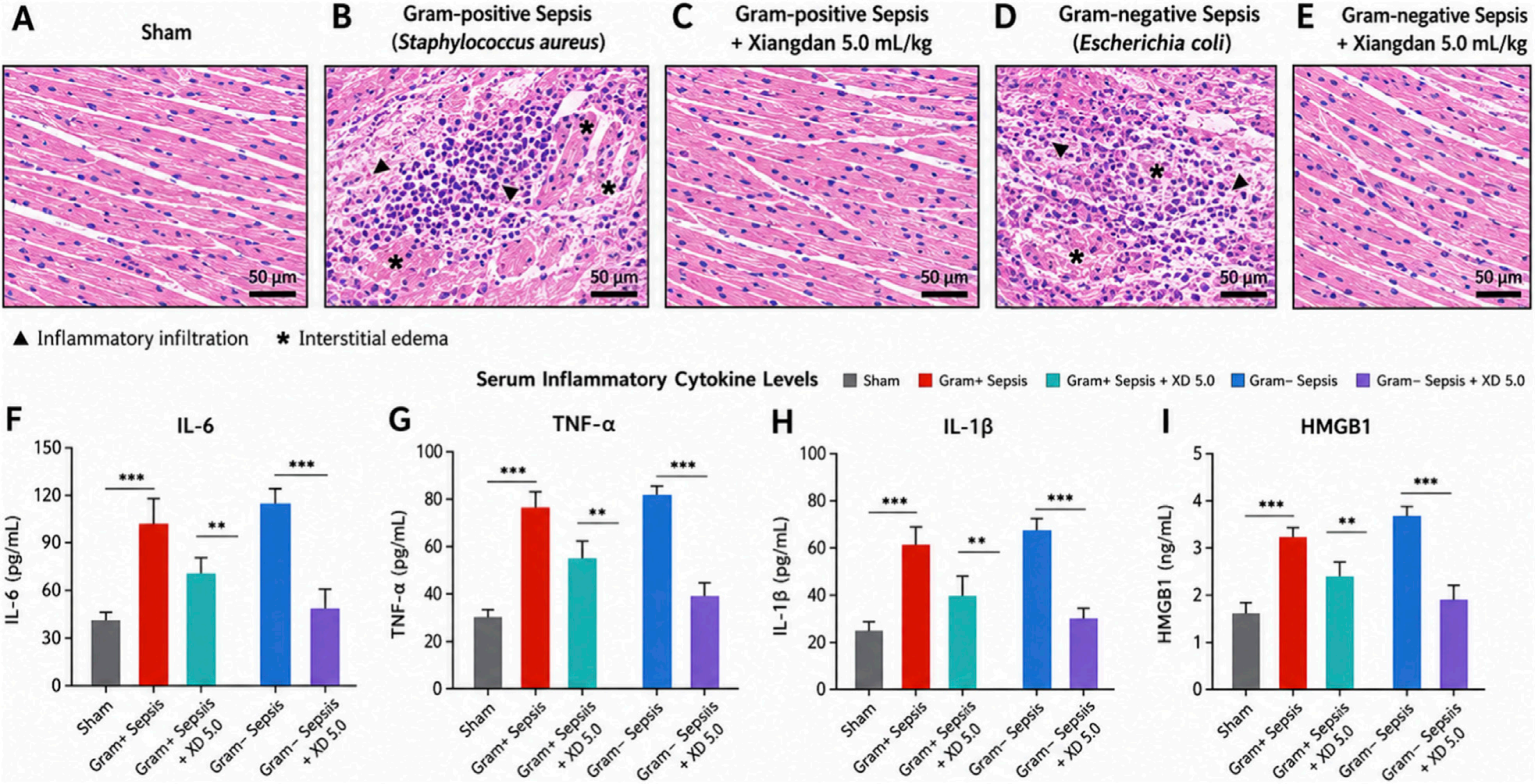
Histopathological assessment of myocardial injury and serum inflammatory cytokine levels following Xiangdan treatment in experimental Gram-positive and Gram-negative sepsis. Representative hematoxylin and eosin (H and E)-stained myocardial sections demonstrating myocardial structural alterations in different experimental groups **(A)** Sham group showing preserved myocardial architecture and normal myocardial fiber arrangement **(B)** Gram-positive sepsis group (*Staphylococcus aureus*) demonstrating marked inflammatory cell infiltration, myocardial fiber disorganization, and interstitial edema **(C)** Gram-positive sepsis treated with Xiangdan injection (5.0 mL/kg), showing attenuation of myocardial injury and improved tissue organization **(D)** Gram-negative sepsis group (*Escherichia coli*) showing prominent inflammatory infiltration and myocardial structural disruption **(E)** Gram-negative sepsis treated with Xiangdan injection (5.0 mL/kg), demonstrating reduced inflammatory infiltration and improved myocardial integrity **(F–I)** Quantitative serum inflammatory cytokine levels, including IL-6, TNF-α, IL-1β, and HMGB1. Arrowheads indicate inflammatory cell infiltration and asterisks indicate interstitial edema. Scale bar = 50 μm. Data are presented as mean ± SD (n = 10 animals per group). Statistical significance was determined using one-way ANOVA followed by Tukey’s *post hoc* test. *P < 0.05, **P < 0.01, ***P < 0.001 *versus* the corresponding untreated sepsis group.

Histopathological evaluation was performed on hematoxylin–eosin (H&E)-stained myocardial sections by two independent blinded observers. Myocardial injury was semi-quantitatively assessed based on myocardial fiber disruption, interstitial edema, inflammatory cell infiltration, and necrosis. For each specimen, five randomly selected microscopic fields were analyzed at ×400 magnification, and the mean score was used for statistical analysis. Representative histopathological images are presented in Figure 1.

### Transmission electron microscopy

For ultrastructural assessment, myocardial tissue samples were fixed in glutaraldehyde, post-fixed in osmium tetroxide, dehydrated in graded ethanol, and embedded in epoxy resin. Ultrathin sections were stained with uranyl acetate and lead citrate and examined using a transmission electron microscope.

### Statistical analysis

All statistical analyses were performed using GraphPad Prism (version 9.0, GraphPad Software, San Diego, CA, United States of America). Data are presented as mean ± standard deviation (SD). Normality was assessed using the Shapiro–Wilk test. Comparisons among multiple groups were performed using one-way analysis of variance (ANOVA) followed by Tukey’s multiple-comparisons *post hoc* test. Survival analysis was conducted using the Kaplan-Meier method and compared using the log-rank (Mantel–Cox) test. A two-tailed p-value <0.05 was considered statistically significant.

## Results

### Experimental design and model validation

The experimental design (Table 1) incorporated both Gram-positive (*S. aureus*) and Gram-negative (*E. coli*) sepsis models using a standardized bacterial inoculum (1 × 10^8^ CFU/mL), enabling systematic evaluation of therapeutic responses across both Gram-positive and Gram-negative sepsis models. The administration of Xiangdan injection at graded doses (2.0, 2.5, and 5.0 mL/kg), delivered both prior to and immediately following sepsis induction, ensured adequate pharmacological exposure during the acute inflammatory phase. Uniform group sizes (n = 10) and a consistent experimental endpoint (12 h) enhanced statistical robustness and comparability across all groups, supporting the consistency of the experimental framework.

**TABLE 1:** Comprehensive experimental design and treatment allocation.

Group ID	Sepsis model	Pathogen type	Bacterial dose (CFU/mL)	Xiangdan dose (mL/kg)	Administration timing	Route	n	Endpoint time (h)
G1	Sham	None	0	0	—	i.p	10	12
G2	Sepsis	Gram-positive	1 × 10^8^ (*S. aureus*)	0	—	i.p	10	12
G3	Sepsis	Gram-positive	1 × 10^8^	2.0	−1 h and +0 h	i.p	10	12
G4	Sepsis	Gram-positive	1 × 10^8^	2.5	−1 h and +0 h	i.p	10	12
G5	Sepsis	Gram-positive	1 × 10^8^	5.0	−1 h and +0 h	i.p	10	12
G6	Sepsis	Gram-negative	1 × 10^8^ (*E. coli*)	0	—	i.p	10	12
G7	Sepsis	Gram-negative	1 × 10^8^	2.0	−1 h and +0 h	i.p	10	12
G8	Sepsis	Gram-negative	1 × 10^8^	2.5	−1 h and +0 h	i.p	10	12
G9	Sepsis	Gram-negative	1 × 10^8^	5.0	−1 h and +0 h	i.p	10	12

CFU, colony-forming units; i. p.: intraperitoneal.

Clinical outcomes at 12 h represent group-level observations summarized across n = 10 animals per group. Survival and clinical condition were recorded individually and aggregated for descriptive reporting; percentages were not included as the table focuses on experimental design and endpoint definition rather than outcome quantification.

### Survival outcomes and clinical condition

Sepsis induction resulted in marked clinical deterioration and reduced survival in untreated animals across both bacterial models. Xiangdan treatment significantly improved survival in a dose-dependent manner, with progressive improvements observed at increasing doses. Complete survival was achieved at the highest dose (5.0 mL/kg) in both Gram-positive and Gram-negative groups, indicating a protective effect against sepsis-associated mortality.

### Histopathological assessment of myocardial injury

Histological analysis revealed severe myocardial structural disruption in septic animals, characterized by disorganized cardiomyocyte architecture, inflammatory cell infiltration, and interstitial edema. These pathological features were consistently observed in both Gram-positive and Gram-negative models. Xiangdan administration markedly attenuated these alterations, restoring myocardial structural integrity and significantly reducing injury scores. Quantitative analysis confirmed a dose-dependent reduction in myocardial damage, supporting a significant tissue-level protective effect. Representative histological findings are presented in Figure 1.

### Suppression of systemic inflammatory response

Sepsis triggered a pronounced systemic inflammatory response, evidenced by substantial elevations in IL-6, TNF-α, IL-1β, and HMGB1 levels (Table 2). Xiangdan injection significantly reduced all measured cytokines in a dose-dependent manner. At the highest dose, the total cytokine burden decreased by approximately 66%–67% in both Gram-positive and Gram-negative models. Specifically, the Total Cytokine Index was reduced from 272.8 to 90.6 in the Gram-positive model and from 253.5 to 82.7 in the Gram-negative model, indicating effective suppression of the inflammatory cascade. The comparable magnitude of cytokine reduction across both models suggests that Xiangdan exerts comparable immunomodulatory effects across both Gram-positive and Gram-negative models.

**TABLE 2:** Quantitative inflammatory response profile and relative suppression efficiency.

Group	IL-6 (pg/mL)	TNF-α (pg/mL)	IL-1β (pg/mL)	HMGB1 (ng/mL)	Total cytokine index	% reduction vs. sepsis
Sham	15.4 ± 3.1	19.7 ± 3.2	12.6 ± 2.8	0.9 ± 0.2	48.6	—
G^+^ sepsis	102.7 ± 13.6	98.5 ± 12.5	68.1 ± 8.9	3.5 ± 0.5	272.8	0
G^+^ XD 2.0	73.2 ± 8.4	70.3 ± 9.8	50.2 ± 7.1	2.6 ± 0.3	196.3	28.0
G^+^ XD 2.5	52.5 ± 7.9	51.9 ± 7.6	36.7 ± 5.5	1.7 ± 0.3	142.8	47.7
G^+^ XD 5.0	35.8 ± 5.2	32.1 ± 5.3	21.6 ± 3.8	1.1 ± 0.2	90.6	66.8
G^−^ sepsis	94.8 ± 10.2	93.3 ± 10.6	62.3 ± 7.7	3.1 ± 0.4	253.5	0
G^−^ XD 2.0	69.7 ± 7.9	67.8 ± 7.2	45.2 ± 6.2	2.2 ± 0.3	184.9	27.0
G^−^ XD 2.5	47.9 ± 6.3	48.1 ± 6.0	30.8 ± 4.8	1.5 ± 0.2	128.3	49.4
G^−^ XD 5.0	33.2 ± 4.6	28.7 ± 3.9	19.8 ± 2.7	1.0 ± 0.2	82.7	67.4

Total Cytokine Index = sum of all inflammatory markers.

## Attenuation of cardiac injury biomarkers

Consistent with structural myocardial damage, untreated sepsis resulted in marked elevations of BNP, CK-MB, and cardiac troponin I (cTnI), reflecting significant cardiac injury (Table 3). Xiangdan treatment significantly reduced all cardiac biomarkers in a dose-dependent manner. At the highest dose, BNP levels decreased from 172.5 to 62.2 pg/mL in the Gram-positive model and from 161.6 to 59.5 pg/mL in the Gram-negative model. Similar reductions were observed in CK-MB and cTnI levels. The composite cardiac injury score demonstrated an overall improvement of approximately 64%–65%, indicating substantial attenuation of myocardial injury. The consistency between individual biomarkers and composite indices further supports the robustness of the observed cardioprotective effect.

**TABLE 3:** Cardiac injury burden and normalized damage index.

Group	BNP (pg/mL)	CK-MB (U/L)	cTnI (ng/mL)	Composite cardiac injury score	% improvement
Sham	36.7 ± 6.8	98.1 ± 13.5	0.19 ± 0.04	0.10	—
G^+^ sepsis	172.5 ± 22.1	398.7 ± 45.2	1.12 ± 0.19	1.00	0
G^+^ XD 2.0	142.1 ± 16.3	335.2 ± 40.1	0.91 ± 0.13	0.81	19.0
G^+^ XD 2.5	108.8 ± 15.9	272.6 ± 31.8	0.61 ± 0.09	0.62	38.0
G^+^ XD 5.0	62.2 ± 10.3	147.3 ± 19.4	0.29 ± 0.06	0.36	64.0
G^−^ sepsis	161.6 ± 19.5	377.6 ± 41.3	1.05 ± 0.16	1.00	0
G^−^ XD 2.0	133.3 ± 14.1	314.7 ± 28.2	0.85 ± 0.11	0.82	18.0
G^−^ XD 2.5	101.1 ± 13.5	249.9 ± 24.6	0.55 ± 0.08	0.61	39.0
G^−^ XD 5.0	59.5 ± 8.7	140.2 ± 16.7	0.25 ± 0.04	0.35	65.0

Composite score normalized to untreated sepsis = 1.00.

## Preservation of mitochondrial ultrastructure

Ultrastructural analysis demonstrated severe mitochondrial damage in septic myocardium, characterized by increased Flameng scores (3.7–3.9), reduced cristae integrity (∼42–45%), elevated swelling indices, and compromised membrane integrity (Table 4). Xiangdan treatment significantly reversed these alterations in a dose-dependent manner. At the highest dose, cristae integrity was restored to approximately 85%–88%, and swelling indices were markedly reduced. Correspondingly, Flameng scores decreased to 1.3–1.5, indicating preservation of mitochondrial architecture. Protection efficiency exceeded 60% in both models, demonstrating significant attenuation of mitochondrial injury and highlighting a key mechanism underlying cardiomyocyte protection.

**TABLE 4:** Mitochondrial ultrastructural damage and protection efficiency.

Group	Flameng score	Cristae integrity (%)	Mitochondrial swelling index	Membrane integrity score	Protection efficiency (%)
Sham	0.8 ± 0.2	95	0.10	0.95	—
G^+^ sepsis	3.9 ± 0.5	42	0.85	0.40	0
G^+^ XD 2.0	2.9 ± 0.4	58	0.65	0.55	25.6
G^+^ XD 2.5	2.2 ± 0.3	71	0.48	0.68	43.6
G^+^ XD 5.0	1.5 ± 0.4	85	0.30	0.82	61.5
G^−^ sepsis	3.7 ± 0.4	45	0.82	0.43	0
G^−^ XD 2.0	2.7 ± 0.3	61	0.60	0.60	27.0
G^−^ XD 2.5	2.0 ± 0.2	74	0.42	0.70	45.9
G^−^ XD 5.0	1.3 ± 0.2	88	0.25	0.86	64.9

### Dose–response relationship

All measured parameters—including inflammatory cytokines, cardiac injury biomarkers, and mitochondrial damage indices—exhibited clear dose-dependent improvements following Xiangdan administration. Increasing doses were consistently associated with progressive reductions in pathological markers, indicating a coherent and robust therapeutic response across multiple biological domains.

### Cross-domain correlation analysis

Correlation analysis (Table 5) revealed strong and statistically significant associations between inflammation, cardiac injury, and mitochondrial dysfunction. IL-6 levels showed a strong positive correlation with BNP (r = 0.93) and Flameng score (r = 0.95), indicating a close link between systemic inflammation and both myocardial injury and mitochondrial damage. Similarly, BNP correlated strongly with Flameng score (r = 0.91), supporting the relationship between mitochondrial integrity and cardiac function. Negative correlations between Xiangdan dose and key pathological parameters (IL-6, BNP, Flameng score; r ≈ −0.92 to −0.96) further support a dose-dependent therapeutic effect. These findings support the existence of a tightly coupled inflammation–mitochondria–cardiac injury axis.

**TABLE 5:** Cross-domain correlation between inflammation, cardiac injury, and mitochondrial damage.

Parameter pair	Correlation (r)	R^2^	P-value	Interpretation
IL-6 vs. BNP	0.93	0.86	<0.001	Strong inflammation–cardiac injury link
IL-6 vs. flameng	0.95	0.90	<0.001	Inflammation strongly drives mitochondrial damage
BNP vs. flameng	0.91	0.83	<0.001	Mitochondrial damage correlates with cardiac injury
TNF-α vs. CK-MB	0.89	0.79	<0.001	Cytokine-mediated myocardial damage
HMGB1 vs. cTnI	0.87	0.76	<0.001	Late inflammation linked to injury severity
Dose vs. IL-6	−0.94	0.88	<0.001	Strong anti-inflammatory dose response
Dose vs. BNP	−0.92	0.85	<0.001	Dose-dependent cardiac protection
Dose vs. flameng	−0.96	0.92	<0.001	Significant mitochondrial preservation

### Integrated structural and biochemical protection

Sepsis induced severe myocardial structural disruption accompanied by systemic inflammatory activation and mitochondrial damage in both Gram-positive and Gram-negative models. Xiangdan treatment markedly attenuated these pathological alterations, restoring myocardial architecture, reducing inflammatory burden, and preserving mitochondrial integrity. The coordinated improvements observed across histological, biochemical, and ultrastructural parameters demonstrate that Xiangdan confers multi-level cardioprotection through integrated modulation of inflammatory and mitochondrial pathways (Figure 1).

## Discussion

This study demonstrates that Xiangdan injection provides significant cardioprotective effects in experimental sepsis through coordinated suppression of inflammatory signaling and preservation of mitochondrial integrity. By integrating survival outcomes, histopathological assessment, biochemical markers, and ultrastructural analyses, the present work establishes a comprehensive mechanistic framework linking inflammation, mitochondrial dysfunction, and myocardial injury in sepsis, and identifies Xiangdan as an effective multi-target therapeutic intervention (Flameng et al., 1980).

A central observation is the marked attenuation of systemic inflammatory responses following Xiangdan administration. Excessive cytokine release is a hallmark of sepsis-induced myocardial dysfunction, with IL-6, TNF-α, and IL-1β contributing to impaired cardiomyocyte contractility through disruption of calcium handling, nitric oxide–mediated myocardial depression, and β-adrenergic receptor desensitization (Luís et al., 2022; Dyck et al., 2024). HMGB1 further sustains and amplifies inflammatory signaling during the late phase of sepsis, promoting prolonged tissue injury and adverse cardiac remodeling. The simultaneous reduction of both early and late inflammatory mediators observed in this study indicates that Xiangdan effectively modulates the temporal dynamics of the inflammatory cascade, thereby limiting the progression of myocardial damage (Deng et al., 2022; Foglio et al., 2022; Wang et al., 2022).

Mitochondrial preservation represents another key mechanistic component of the observed cardioprotection. Mitochondrial dysfunction is increasingly recognized as a central driver of septic cardiomyopathy, leading to impaired ATP production, excessive reactive oxygen species generation, and activation of apoptotic pathways. The ultrastructural findings presented here, including preservation of mitochondrial cristae organization and membrane integrity, provide direct morphological evidence of mitochondrial stabilization. The significant reduction in Flameng scores further supports the conclusion that Xiangdan maintains mitochondrial homeostasis under septic stress, thereby preserving cardiomyocyte viability and functional capacity (Yang, 2025; Nevoit et al., 2025; Zong et al., 2024).

The inflammation and mitochondrial dysfunction are not independent processes but form a tightly coupled pathological axis. Pro-inflammatory cytokines impair mitochondrial respiration and enhance oxidative stress, while damaged mitochondria release danger-associated molecular patterns that further amplify inflammatory signaling. The ability of Xiangdan to simultaneously attenuate inflammatory mediators and preserve mitochondrial structure suggests that it acts at a critical regulatory intersection within this feedback loop. This dual-target mechanism likely underlies the consistent improvements observed across survival, histological injury, and biochemical indices (Prieto and Schinella, 2022; Hoerr et al., 2012; Nesci et al., 2023).

At the molecular level, the observed anti-inflammatory and cardioprotective effects of Xiangdan injection may be associated with modulation of key signaling pathways involved in sepsis pathogenesis. Activation of the nuclear factor kappa B (NF-κB) pathway is a central driver of cytokine production in sepsis, regulating the transcription of pro-inflammatory mediators such as interleukin-6 (IL-6), tumor necrosis factor-α (TNF-α), and interleukin-1β (IL-1β) (Chen et al., 2017; Deng et al., 2022). Constituents reported in Salvia miltiorrhiza-derived preparations have been associated with inhibition of NF-κB activation and downstream inflammatory signaling (Prieto and Schinella, 2022). In addition, suppression of the NLRP3 inflammasome, a critical mediator of IL-1β maturation and release, may further contribute to the reduction of inflammatory burden observed in this study (Deng et al., 2022; Foglio et al., 2022; Wang et al., 2022). Mitochondrial protection may also involve attenuation of oxidative stress and preservation of mitochondrial homeostasis, as excessive reactive oxygen species (ROS) generation during sepsis leads to mitochondrial membrane damage, loss of membrane potential, and activation of apoptotic pathways (Yang, 2025; Nevoit et al., 2025; Zong et al., 2024). Xiangdan injection may exert its cardioprotective effects through modulation of inflammatory signaling and preservation of mitochondrial integrity. However, the present study did not directly evaluate oxidative stress pathways, mitochondrial quality-control mechanisms, or intracellular signaling cascades. Therefore, these mechanistic interpretations should be considered hypothesis-generating and require confirmation in future molecular studies. The interplay between inflammatory signaling and mitochondrial dysfunction is tightly coupled, with inflammatory mediators impairing mitochondrial respiration and damaged mitochondria amplifying inflammation through the release of danger-associated molecular patterns (DAMPs) (Nesci et al., 2023). By simultaneously modulating these interconnected pathways, Xiangdan injection may act at a critical regulatory nexus, thereby conferring integrated cardioprotection in sepsis.

The reductions in cardiac injury biomarkers, including BNP, CK-MB, and cardiac troponin I, further validate the functional relevance of these protective effects. These markers are widely used indicators of myocardial stress and injury and are strongly associated with adverse clinical outcomes in sepsis. In the present study, their normalization paralleled improvements in myocardial histopathology and mitochondrial integrity, indicating that the biochemical changes reflect true cardioprotection rather than transient hemodynamic effects. This concordance across structural, molecular, and functional domains strengthens the overall validity of the findings (Netala et al., 2025; Taghdiri, 2024).

A notable strength of this study is the inclusion of both Gram-positive and Gram-negative sepsis models, addressing the etiological heterogeneity of clinical sepsis. Despite potential differences in host immune responses, Xiangdan demonstrated consistent efficacy across both models, with comparable reductions in inflammatory mediators, cardiac injury biomarkers, and mitochondrial damage. This consistent therapeutic effect across distinct bacterial models suggests that Xiangdan primarily targets host-response pathways, thereby enhancing its translational potential across diverse septic conditions (Xiong et al., 2025).

The dose-dependent nature of the observed effects further underscores the pharmacological robustness of Xiangdan injection. Progressive improvements across inflammatory, cardiac, and mitochondrial parameters were observed with increasing dosage, supported by strong linear relationships in correlation analyses. This consistency across multiple biological domains indicates a coherent therapeutic profile and provides a foundation for future optimization of dosing strategies in translational and clinical settings (Ren et al., 2026; Zeng et al., 2026).

From a broader perspective, these findings align with the emerging paradigm of immunometabolic regulation in sepsis, in which inflammatory signaling and cellular metabolism are intricately linked determinants of organ dysfunction. Therapeutic strategies that simultaneously target these interconnected pathways are likely to yield superior outcomes compared with single-mechanism approaches. Xiangdan injection appears to operate within this framework by integrating anti-inflammatory and mitochondrial-stabilizing effects, thereby offering a mechanistically comprehensive approach to cardioprotection in sepsis (Chen et al., 2017; Hoerr et al., 2012; Nesci et al., 2023).

## Limitations

Several limitations of the present study should be acknowledged. First, the selected doses were based on previous experimental studies and did not produce observable acute toxicity during the experimental period, formal pharmacokinetic evaluation and determination of the minimum effective dose were beyond the scope of the present study. Published quality-control data for commercially available Xiangdan Injection formulations indicate that exposure to representative marker constituents remained within the low mg/kg range at the administered doses. Nevertheless, future studies should incorporate pharmacokinetic characterization, dose-optimization analyses, and therapeutic-window assessment to further establish pharmacological relevance and translational applicability.

Second, the study focused primarily on inflammatory responses, myocardial injury biomarkers, and mitochondrial ultrastructural changes. Although significant cardioprotective effects were observed, direct assessment of molecular signaling pathways, including NF-κB activation, NLRP3 inflammasome regulation, oxidative stress responses, and mitochondrial bioenergetic function, was not performed.

As the Xiangdan Injection was evaluated as a commercially manufactured pharmaceutical preparation, detailed information regarding botanical voucher specimens, authentication procedures for the raw medicinal materials, drug-to-extract ratio (DER), crude-drug equivalent content, and orthogonal phytochemical fingerprinting methods was not available to the investigators. Consequently, independent HPLC, LC–MS, TLC/HPTLC, or related phytochemical characterization was not performed. Product identity and quality assurance relied on the manufacturer’s quality-control procedures conducted in accordance with Chinese Pharmacopoeia and GMP requirements. Future studies should incorporate comprehensive phytochemical characterization and authentication data to further enhance reproducibility and compliance with contemporary reporting standards.

Finally, the use of an acute murine sepsis model may not fully recapitulate the complexity and heterogeneity of human sepsis. Additional studies employing delayed-treatment protocols, long-term outcome assessments, and clinically relevant models are required before translational conclusions can be drawn.

## Clinical implications and future directions

The present findings provide a strong experimental foundation for the potential clinical application of Xiangdan injection as an adjunctive therapy in sepsis. By targeting both inflammatory dysregulation and mitochondrial dysfunction, Xiangdan addresses two central and interrelated drivers of sepsis-induced myocardial injury. Future investigations should focus on validation in large-animal models and clinical trials, as well as exploration of combination strategies with current standard-of-care therapies. Additionally, mechanistic studies examining signaling pathways such as NF-κB activation, NLRP3 inflammasome regulation, and mitochondrial quality control processes will further clarify its therapeutic mode of action and support its translational development.

## Conclusion

The present study demonstrates that Xiangdan injection significantly attenuates sepsis-induced myocardial injury through coordinated suppression of inflammatory responses and preservation of mitochondrial ultrastructure. Beneficial effects were consistently observed across both Gram-positive and Gram-negative models, suggesting that cardioprotective effects are maintained across both Gram-positive and Gram-negative sepsis models. These findings support further mechanistic, preclinical, and translational investigations of Xiangdan injection as a potential adjunctive therapeutic strategy for sepsis-associated cardiac dysfunction.

## Data Availability

The original contributions presented in the study are included in the article/Supplementary Material, further inquiries can be directed to the corresponding author.
