# Supplementary material for: Xiangdan Injection attenuates experimental sepsis-induced myocardial injury by suppressing inflammation and preserving mitochondrial ultrastructure
Source: Front Pharmacol. 2026 Aug 4;17:1860982. doi: 10.3389/fphar.2026.1860982 (PMC13481551; doi:10.3389/fphar.2026.1860982)
Supplement: Supplementary file 1 [file DataSheet1.pdf]

Table S1. ConPhyMP Table 1: Description of Botanical Materials and Product Information

Section	Item	Information Reported
Product Information	Product name	Xiangdan Injection (Xiangdan Zhusheye, 香丹注射液)
Product Information	Product category	Commercial sterile injectable traditional Chinese medicine preparation
Product Information	Manufacturer	Guangzhou Baiyunshan Mingxing Pharmaceutical Co., Ltd., Guangzhou, China
Product Information	Pharmaceutical form	Sterile injectable solution
Product Information	Regulatory status	Commercial pharmaceutical preparation manufactured according to Chinese Pharmacopoeia standards
Product Information	Manufacturing standard	Good Manufacturing Practice (GMP)
Botanical Material	Botanical source 1	*Salvia miltiorrhiza* Bunge
Botanical Material	Family	Lamiaceae
Botanical Material	Pharmacopoeial name	*Salviae miltiorrhizae radix et rhizoma*
Botanical Material	Plant part used	Root and rhizome
Botanical Material	Botanical source 2	*Dalbergia odorifera* T.C.Chen
Botanical Material	Family	Fabaceae
Botanical Material	Pharmacopoeial name	*Dalbergiae odoriferae lignum*
Botanical Material	Plant part used	Heartwood
Authentication	Botanical authentication	Performed by manufacturer according to Chinese Pharmacopoeia quality-control specifications
Authentication	Voucher specimen	Quality documentation maintained by manufacturer
Authentication	Identification method	Pharmacognostic identification and quality-control testing according to Chinese Pharmacopoeia standards
Collection Information	Collection locality	Not disclosed by manufacturer
Collection Information	Harvest season	Not disclosed by manufacturer
Extraction Information	Extraction solvent	Proprietary manufacturing process

Extraction Information	Extraction method	Proprietary manufacturing process
Extraction Information	Drug-to-solvent ratio (DSR)	Not disclosed by manufacturer
Extraction Information	Drug-to-extract ratio (DER)	Not disclosed by manufacturer
Extraction Information	Further purification	Manufacturer quality-controlled production process
Quality Control	Quality standard	Chinese Pharmacopoeia specifications
Quality Control	Batch-release testing	Conducted by manufacturer
Storage	Storage conditions	Stored according to manufacturer recommendations
Experimental Use	Additional extraction by investigators	None
Experimental Use	Additional fractionation by investigators	None
Experimental Use	Additional purification by investigators	None
Experimental Use	Material used in study	Finished pharmaceutical product used directly
Experimental Use	Route used in study	Intraperitoneal administration
Experimental Use	Purpose	Evaluation of cardioprotective effects in experimental sepsis

Table S2. ConPhyMP Table 2a: Extract Characterization and Analytical Information

ConPhyMP Item	Information
Extract classification	Type A extract (commercial pharmacopoeial herbal medicinal preparation)
Product name	Xiangdan Injection (香丹注射液)
Manufacturer	Guangzhou Baiyunshan Mingxing Pharmaceutical Co., Ltd., Guangzhou, China
Botanical ingredients	*Salvia miltiorrhiza* Bunge and *Dalbergia odorifera* T.C.Chen
Quality standard	Chinese Pharmacopoeia
Manufacturing standard	GMP-compliant production
Product source	Commercial pharmaceutical preparation
Identity verification	Performed by manufacturer according to pharmacopoeial requirements
Botanical authentication	Performed by manufacturer according to Chinese Pharmacopoeia standards
Batch consistency	Controlled through manufacturer quality-assurance and batch-release procedures
Purity assessment	Conducted according to manufacturer quality-control standards
Safety assessment	Conducted according to pharmaceutical manufacturing regulations
Active constituents	Multiple bioactive constituents derived from *Salvia miltiorrhiza* and *Dalbergia odorifera*
Marker compounds	Pharmacopoeial quality-control markers maintained by manufacturer
Independent phytochemical fingerprinting	Not performed in current study
Independent HPLC fingerprint	Not performed
Independent LC–MS analysis	Not performed
Independent TLC/HPTLC analysis	Not performed
Independent marker quantification	Not performed
Additional extraction by investigators	Not applicable
Additional fractionation by investigators	Not applicable
Additional purification by investigators	Not applicable
Material evaluated	Finished commercial pharmaceutical product
Experimental objective	Pharmacological evaluation of sepsis-induced myocardial injury
Compliance with ConPhyMP	Botanical origin, manufacturer information, pharmaceutical status, and quality-control framework reported

Study limitation	Detailed proprietary manufacturing specifications and analytical fingerprints were not available because a finished commercial pharmaceutical product was investigated

Xiangdan Injection was investigated as a finished commercial pharmaceutical preparation. Manufacturing information, botanical authentication, and quality-control procedures were provided through the manufacturer and Chinese Pharmacopoeia standards. Independent phytochemical characterization was beyond the scope of the present study.
